# Dicer-Dependent Biogenesis of Small RNAs and Evidence for MicroRNA-Like RNAs in the Penicillin Producing Fungus *Penicillium chrysogenum*

**DOI:** 10.1371/journal.pone.0125989

**Published:** 2015-05-08

**Authors:** Tim A. Dahlmann, Ulrich Kück

**Affiliations:** Christian Doppler Laboratory for “Fungal Biotechnology”, Lehrstuhl für Allgemeine und Molekulare Botanik, Ruhr-Universität Bochum, Universitätsstr. 150, D-44780 Bochum, Germany; Cinvestav, MEXICO

## Abstract

MicroRNAs (miRNAs) are non-coding small RNAs (sRNAs) that regulate gene expression in a wide range of eukaryotes. In this study, we analyzed regulatory sRNAs in *Penicillium chrysogenum*, the industrial producer of the β-lactam antibiotic penicillin. To identify sRNAs and microRNA-like RNAs (milRNAs) on a global approach, two sRNA sequencing libraries were constructed. One library was created with pooled total RNA, obtained from twelve differently grown cultures (RNA Mix), and the other with total RNA from a single submerged cultivation (∆*ku70*FRT2). Illumina sequencing of both RNA libraries produced 84,322,825 mapped reads. To distinguish between Dicer-dependent and independent sRNA formation, we further constructed two single *dicer* gene mutants (∆*dcl2* and ∆*dcl1*) and a *dicer* double mutant (∆*dcl2*∆*dcl1*) and analyzed an sRNA library from the Dicer-deficient double-mutant. We identified 661 Dicer-dependent loci and *in silico* prediction revealed 34 milRNAs. Northern blot hybridization of two milRNAs provided evidence for mature milRNAs that are processed either in a complete or partial Dicer-dependent manner from an RNA precursor. Identified milRNAs share typical characteristics of previously discovered fungal milRNAs, like a strong preference for a 5' uracil and the typical length distribution. The detection of potential milRNA target sites in the genome suggests that milRNAs might play a role in posttranscriptional gene regulation. Our data will further increase our knowledge of sRNA dependent gene regulation processes, which is an important prerequisite to develop more effective strategies for improving industrial fermentations with *P*. *chrysogenum*.

## Introduction

Small regulatory RNAs are short non-coding RNAs ranging in size from 17 to 29 nt that mediate RNA interference (RNAi). Small RNAs (sRNAs) have been discovered in most eukaryotic organisms, and can be divided into three major regulatory classes [1–3]: piwi-interacting RNAs (piwiRNAs), short interfering RNAs (siRNAs), and microRNAs (miRNAs). Their endogenous or extrinsic origins and different biogenesis pathways can distinguish these sRNAs from each other. SiRNAs and miRNAs are usually derived from double stranded RNAs (dsRNAs) that are processed by Dicer-like proteins, while piwiRNAs originate Dicer-independently from a single stranded RNA (ssRNA) [4,5]. In contrary to siRNAs, which are derived from double-stranded RNA molecules, miRNAs are processed from a nuclear-encoded single-stranded precursor RNA that forms a typical RNA stem-loop structure [6]. Besides exogenous siRNAs that mediate silencing of selfish genetic elements, endogenous siRNAs have been reported that regulate expression of endogenous genes in animals, plants, and fungi. These siRNAs originate from exonic regions (exonic-siRNAs), and target the mRNAs of protein coding genes from which they were produced [5,7–9].

Investigations into the Quelling phenomenon in *Neurospora crassa* led to the early discovery of regulatory sRNAs in a fungal organism [10]. This phenomenon is the silencing of multiple copies of homologous sequences, which were integrated by DNA transformation into fungal genomic DNA. In these cases, additional copies of the transgene induced the RNAi silencing machinery and siRNAs were processed by Dicer-like enzymes from dsRNAs generated by RNA-dependent RNA polymerases (RdRP) [11]. While the phenomenon of Quelling is well understood at the molecular level, little is known so far about fungal miRNAs. The first fungal microRNA-like RNAs (milRNAs) were discovered in *N*. *crassa* [12]. MilRNAs have also been detected in other fungal organisms, such as *Cryptococcus neoformans*, *Sclerotinia sclerotiorum*, *Trichoderma reseei*, *Fusarium oxysporum*, and *Penicillium marneffei* but their regulatory impact on gene expression and the mechanism of milRNA-target interaction is poorly understood [13–17]. In plants, near-perfect base pairing between the miRNA and its target is necessary, and results in direct cleavage of the target mRNA. In stark contrast, target recognition in animals requires a less stringent 7–8 nt long seed sequence at the 5’ end of the miRNA [18]. Besides directly degrading mRNA, animal and plant miRNAs can mediate suppression of mRNA translation [19,20]. Analysis of milRNAs from *N*. *crassa* and *C*. *neoformans* showed that milRNAs negatively regulate protein expressions of reporter genes, which contain perfect complementary target sites [12,13]. Nevertheless, the mechanisms involved in targeted gene silencing by fungal milRNAs are mostly unknown, and so far little information about target recognition is available.

Fungal milRNAs share many similarities with animal and plant miRNAs. All are similarly processed from stem-loop RNA precursors and the majority require a Dicer-like enzyme for their biogenesis [21]. In addition, Argonaute-like proteins, a core component of the RNA-induced silencing complex (RISC), are necessary to trigger gene silencing. Interestingly, the core components of RISC are highly conserved in diverse eukaryotes including fungi. With the exception of Saccharomycotina, the majority of ascomycetes analyzed so far contain at least one pair of Dicer-like and Argonaute-like protein coding genes [22,23].

Here, we analyzed sRNAs from *Penicillium chrysogenum*, the industrial producer of the β-lactam antibiotic penicillin. Previously, we showed that the genome contains two Dicer-like proteins [24]. Furthermore, a large (>200 nt) perfectly complementary artificial hairpin structure is processed by Dicer-like protein Dcl2 to generate functional siRNAs [24]. To detect milRNAs in *P*. *chrysogenum*, we analyzed different sRNA sequencing libraries from the industrial production strain P2niaD18, laboratory strain ∆*ku70*FRT2, lacking the Ku70 polypeptide of the non-homologous end joining (NHEJ) complex, and a Dicer-deficient mutant (∆*dcl2*∆*dcl1*) [25,26]. Sequencing of the small RNA transcriptome, followed by bioinformatic analyses to predict milRNAs and their putative targets, provide unequivocal evidence for Dicer-dependent sRNAs and milRNAs in *P*. *chrysogenum*.

## Results

### Analysis of small RNA libraries

For a comprehensive survey of sRNAs from *P*. *chrysogenum*, low molecular weight RNA (18–50 nt) was isolated and used for small RNA sequencing. For RNA isolation, the industrial laboratory strain P2niaD18 was grown on four different media and harvested at three different time points (see Material and Methods). From these 12 differently grown cultures, RNA was isolated and pooled to generate a sample designated “RNA-Mix”. Two further small RNA libraries were generated from submerged cultures of ∆*ku70*FRT2 and Dicer-double mutant ∆*ku70*FRT2∆*dcl2*∆*dcl1*, hereafter referred to as ∆*dcl2*∆*dcl1*. After removing low quality reads and trimming of 3'- adaptor sequences, 46,240,082, 52,144,415, and 65,660,252 trimmed reads were obtained as "total" read datasets for the RNA-Mix, ∆*ku70*FRT2 and ∆*dcl2*∆*dcl1*. For subsequent analysis, the number of identical reads within each total dataset was calculated and all total reads were collapsed and transferred to a "unique" dataset. To avoid accumulation of rare reads, such as reads derived from degradation products, only unique reads with a read count of at least 10 were used for further analyses.

Total and filtered unique reads were mapped to the reference genome of P2niaD18 [25]. For the RNA-Mix, we found that 84.8% of all total reads (39,188,594) map at least once to the reference genome. Identical reads were combined to form a set of 1,117,770 unique reads and of these 122,018 were represented with a minimal read count of 10. The corresponding values of total reads for ∆*ku70*FRT2 and ∆*dcl2*∆*dcl1* that mapped at least once are 86.6% (45,134,231) and 87.9% (57,721,023) respectively. Within these samples we found 1,457,875 and 1,290,420 unique reads, respectively, including 113,603 and 104,922 unique reads with read numbers equal or higher than 10 (Table 1).

**Table 1:** Statistical summary of small RNA sequencing data and distribution of small RNAs with perfect match to the genome sequence of *P*. *chrysogenum*.

		Total reads			Unique reads (≥ 10 reads)		
RNAs (15–36 nt)		RNA-Mix	∆*ku70*FRT2	∆*dcl2*∆*dcl1*	RNA-Mix	∆*ku70*FRT2	∆*dcl2*∆*dcl1*
**Raw reads**		46,240,082	52,144,415	65,660,252	122,018	113,603	104,922
**Mapped reads^[^^1^^]^**		39,188,594	45,134,231	57,721,023	89,096	77,035	67,428
**Ratio**		84.80%	86.60%	87.90%	73.01%	67.81%	64.26%
**Protein-coding genes**		16,171,567	16,249,031	20,055,379	38,337	35,173	26,558
	sense	11,612,935	10,975,391	13,497,944	29,659	27,835	24,798
	antisense	4,942,727	5,554,209	6,874,230	10,389	8,631	2,679
**Exon**		5,619,395	6,472,049	7,539,001	16,527	17,680	8,431
	sense	1,633,831	1,687,351	1,557,211	9,788	11,875	7,596
	antisense	4,009,410	4,799,719	5,984,778	7,043	6,020	865
**Intron**		10,569,963	9,777,092	12,504,834	21,702	17,439	18,022
	sense	9,996,408	9,276,048	11,918,300	19,744	15,839	17,085
	antisense	905,029	741,304	873,060	3,238	2,557	1,729
**tRNA**		6,128,717	6,746,039	8,779,724	5,062	4,133	4,363
	sense	6,126,353	6,745,245	8,779,649	5,025	4,123	4,363
	antisense	2,577	971	277	38	11	1
**rRNA**		22,659,643	29,792,848	39,201,820	43,143	34,808	39,639
	sense	22,652,991	29,780,783	39,201,243	43,051	34,639	39,626
	antisense	6,652	12,065	577	92	169	3
**Intergenic**		21,367,882	27,127,757	35,564,101	50,226	41,195	35,646

^[1]^ Perfectly mapped reads with more than one locus may occur in several categories as mapped reads.

**Fig 1:**
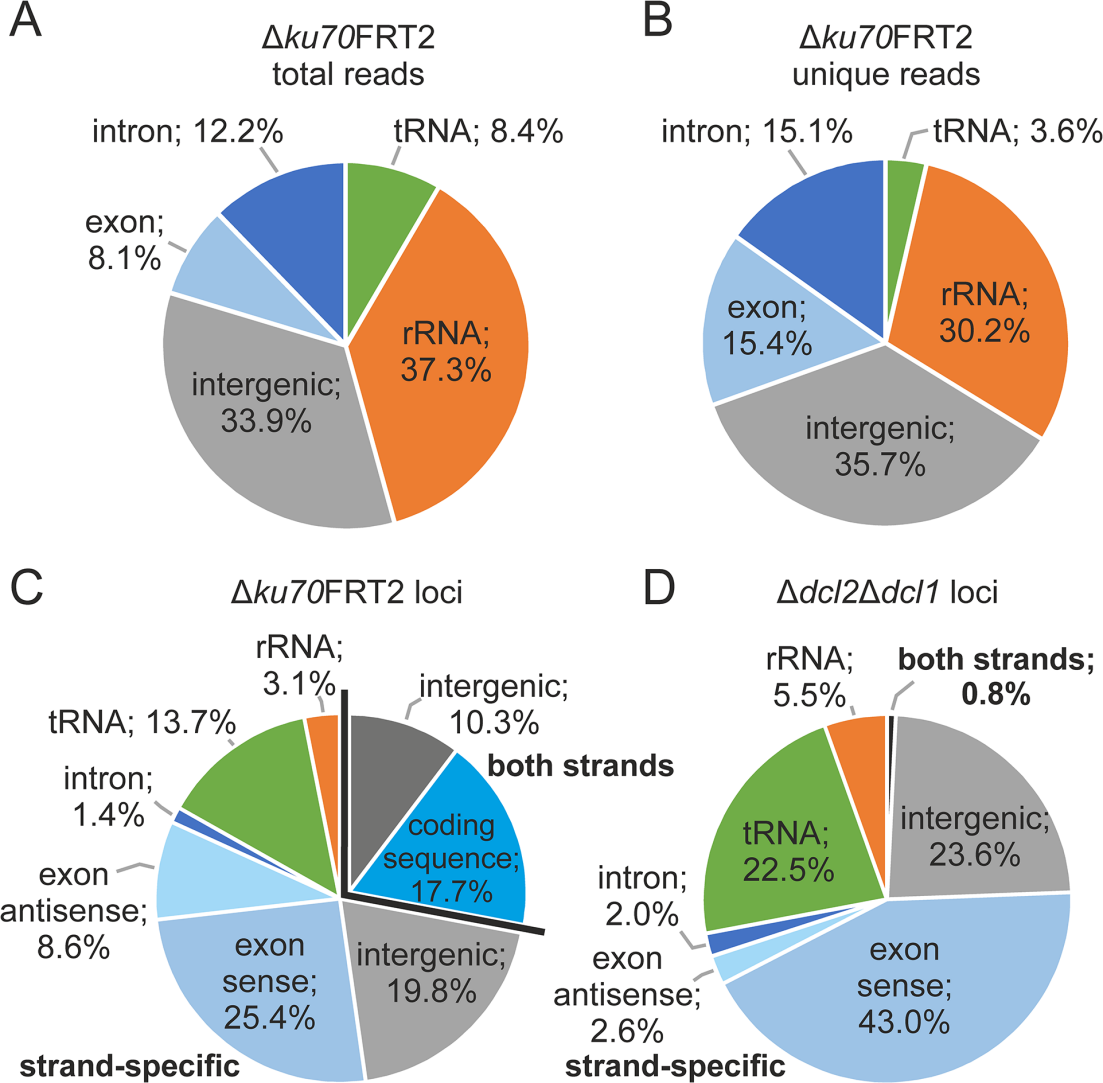
Chromosomal distribution of small RNAs and sRNA-producing loci. Pie graphs for total reads (A) and unique reads (B) are showing the relative abundance of sRNAs located in tRNAs, rRNAs, intergenic, exonic and intronic regions in ∆*ku70*FRT2. Alignments of sRNA-producing loci of ∆*ku70*FRT2 (C) and ∆*dcl2*∆*dcl1* (D) show that the number of sRNAs that map to both DNA strands of one feature have strongly increased and that the fraction of sRNA loci that align to exonic regions in sense orientation has decreased substantially in ∆*dcl2*∆*dcl1* compared to ∆*ku70*FRT2.

To determine the origin of the sRNAs, we mapped the sequence data acquired from the RNA-Mix, ∆*ku70*FRT2, and ∆*dcl2*∆*dcl1* to intergenic, exonic, and intronic regions and to rRNA and tRNA sequences. The proportion of reads that mapped to intragenic and intergenic regions, as well as tRNA and rRNA genes looked highly similar for all three libraries (S1 Fig) and is exemplarily displayed for ∆*ku70*FRT2 in Fig 1A and 1B. Within the total dataset, degraded rRNAs represented with 37.3% a rather high proportion. 33.9% of total reads mapped to intergenic regions and the amount of reads that mapped to introns (12.2%), exons (8.1%), and tRNAs (8.4%) was relatively low (Fig 1A). Interestingly, the number of total reads mapping forward to exonic sequences was lower than the number originating from the antisense sequences of these coding sequences (Table 1). Within the unique dataset of ∆*ku70*FRT2, the read distribution changed towards a higher fraction of reads mapping to exonic sequences (15.4%), while the percentage of reads from rRNA and tRNA regions declined to 30.2% and 3.6% (Fig 1B). To identify sRNA-producing loci, overlapping and adjacent unique reads were grouped. 1,693 loci were calculated for ∆*ku70*FRT2 and 783 for ∆*dcl2*∆*dcl1*. Loci were mapped to the P2niaD18 genome and allocation of the loci to annotated genomic regions was performed. We calculated 1319 sRNA-producing loci in ∆*ku70*FRT2 and 374 of these generated sRNAs in forward and reverse orientation from the same genomic region. In ∆*dcl2*∆*dcl1*, 783 sRNA-producing loci were identified but only 6 loci yielded sRNAs of both orientations and were located in intergenic regions or rRNA genes (Table 2). Pie graphs illustrate that the proportion of loci that map to different features has significantly been altered between ∆*ku70*FRT (Fig 1C) and the Dicer-deficient strain (Fig 1D). The percentage of sRNA-producing loci in exonic regions in sense orientation and the number of loci mapping to tRNAs and rRNAs genes have strongly increased in ∆*dcl2*∆*dcl1* compared to ∆*ku70*FRT. In particular, the significant depletion of sRNA-producing loci with sRNAs that map in both orientations is noteworthy and points to the fact that Dicer-dependent sRNAs in *P*. *chrysogenum* are mostly produced from coding sequences and intergenic regions.

**Table 2:** Characterization of small RNA producing loci.

		RNA-Mix	∆*ku70*FRT2	∆*dcl2*∆*dcl1*
**total number of sRNA-loci**		1513	1319	783
**loci with sRNAs on both strands**		438	374	6
**sRNA appearance**				
**Exon**	on both strands	292	233	0
	only sense	319	335	337
	only antisense	137	114	20
**Intron**	on both strand	2	1	0
	strand-specific	21	17	16
**tRNA**	on both strands	4	0	0
	strand-specific	177	181	176
**rRNA**	on both strands	3	4	3
	strand specific	37	37	43
**Intergenic**	on both strands	137	136	3
	strand-specific	384	261	185

### Identification and characterization of Dicer-dependent small RNAs

Previously, we showed that a mutant lacking the gene coding for Dcl2 had a wild-type morphology, although siRNAs are not processed [24]. To identify Dicer-dependent sRNAs, read counts obtained from the ∆*ku70*FRT and ∆*dcl2*∆*dcl1* were normalized and compared. Furthermore, we characterized the identified sRNAs for specific traits of sRNAs, such as origin, length distribution, and starting nucleotide preference.

**Fig 2:**
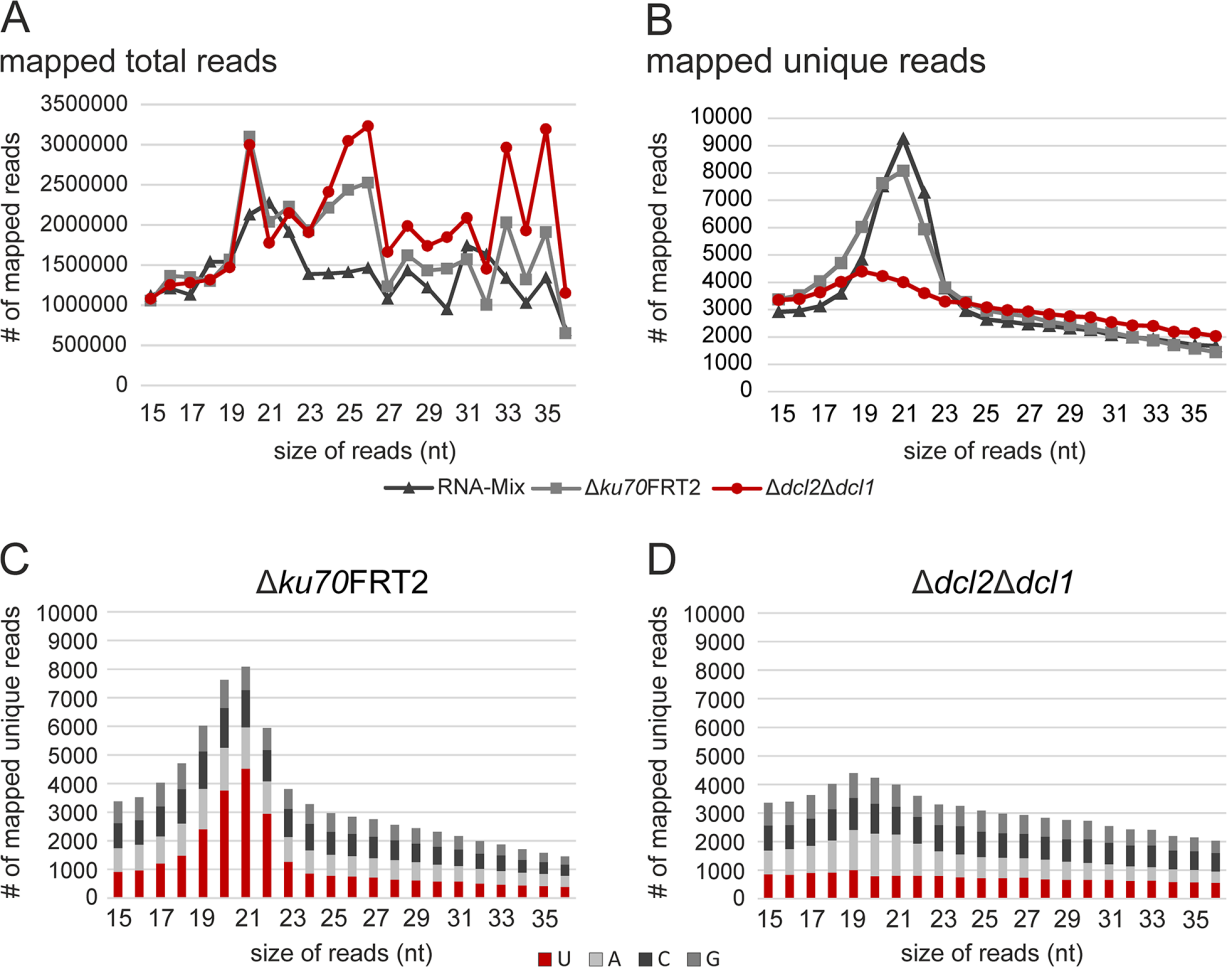
Length distribution of *P*. *chrysogenum* small RNA population. Length distribution of mapping sRNA reads for the datasets of total (A) and unique (B) reads obtained from three different samples. Frequency of the 5'-nucleotide of the unique reads of ∆*ku70*FRT2 (C) and ∆*dcl2*∆*dcl1* (D) in dependency of their read length.

Length distributions of mapped reads ranging from 15 to 36 nt within the total and unique datasets are displayed in Fig 2A and 2B. Although the total reads were more or less irregularly distributed for all three samples, unique reads showed a significant peak at 21 nt for RNA-Mix and ∆*ku70*FRT2, but not for ∆*dcl2*∆*dcl1*. In addition, we determined the first nucleotide of all unique reads. Recent reports have indicated a preference for regulatory sRNAs to start with uracil [12,27]. As expected for Dicer-processed sRNAs, unique reads obtained from RNA-Mix and ∆*ku70*FRT2 had an increased ratio of reads starting with uracil (58.0% and 49.2%). In ∆*dcl2*∆*dcl1* (20.9%), this phenomenon is not observed, obviously due to the absence of both Dicer-like proteins. To further demonstrate that the accumulation of reads starting with uracil depends on Dicer processing, all unique reads were calculated separately according to their read lengths. In ∆*ku70*FRT2, reads starting with uracil were predominantly found in the range between 18 to 23 nt, which is a common length of Dicer-processed sRNAs (Fig 2C) [12]. In contrary, no accumulation of reads with a 5'-uracil was present in ∆*dcl2*∆*dcl1* (Fig 2D).

**Fig 3:**
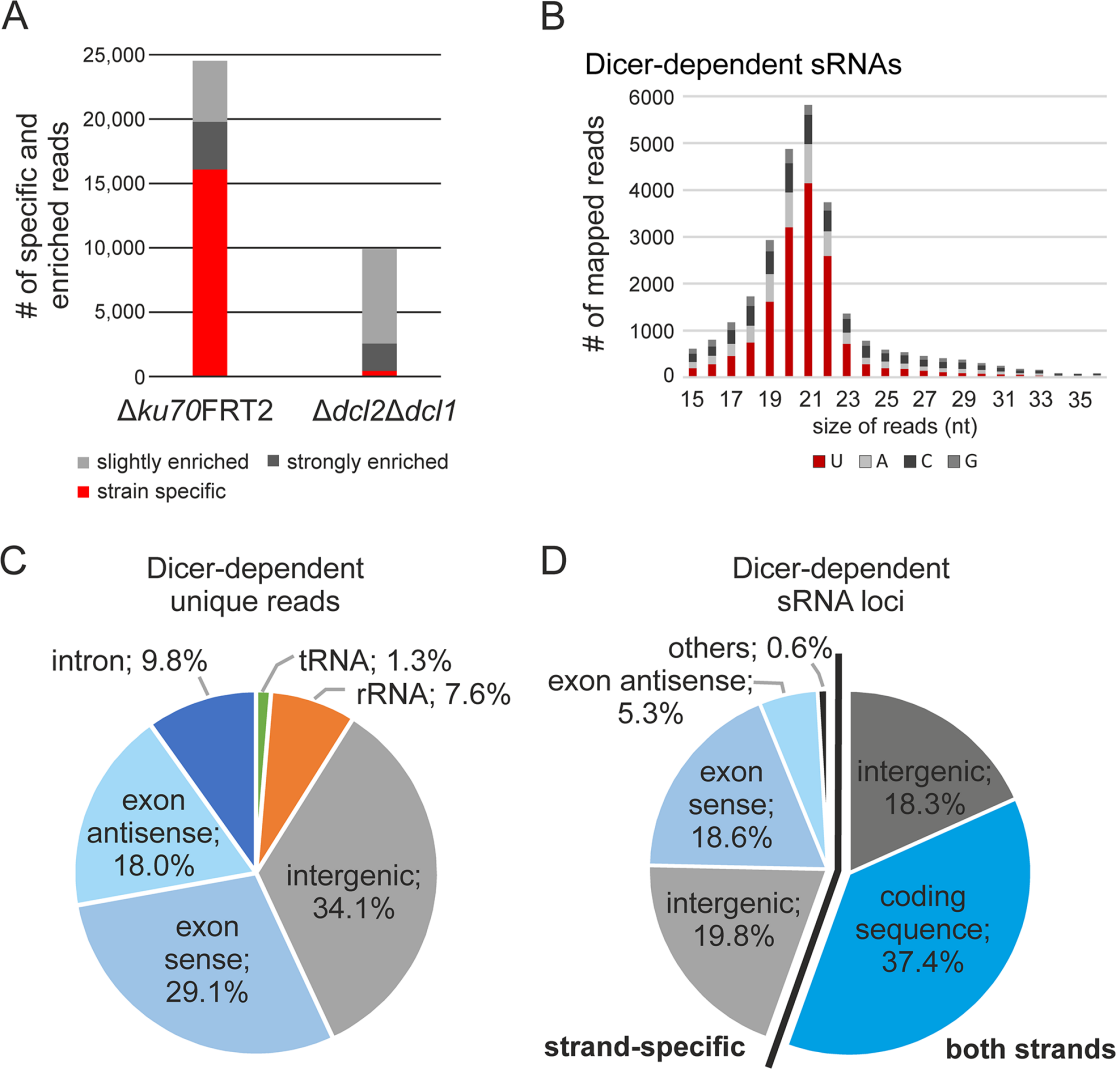
Characterization of Dicer-dependent small RNAs. (A) Strain-specific and overrepresented unique reads in ∆*ku70*FRT2 compared to ∆*dcl2*∆*dcl1* and *vice versa*. (B) Nucleotide preference and size distribution of Dicer-dependent small RNAs. (C) Pie graphs of the relative abundance of Dicer-dependent sRNAs and (D) Dicer-dependent sRNA-producing loci in accordance to their strand bias.

To identify Dicer-dependent sRNAs, all unique sRNAs in ∆*ku70*FRT2 were compared to those found in ∆*dcl2*∆*dcl1*. After normalization of the datasets we identified 16,090 unique reads from ∆*ku70*FRT2 that were absent in the total read dataset of ∆*dcl2*∆*dcl1*. Further, we found 3,695 unique reads that are strongly (log_2_≤-2) and 4,734 that are slightly (log_2_ between -2 and -1) underrepresented in ∆*dcl2*∆*dcl1* compared to ∆*ku70*FRT2. On the contrary, the same analysis of the unique reads of ∆*dcl2*∆*dcl1* resulted in only 440 different reads that were not present in ∆*ku70*FRT2 (Fig 3A). The overwhelming majority of Dicer-dependent unique reads had a length between 17 and 23 nt and 64.8% start with uracil (Fig 3B), while Dicer-independent sRNAs (∆*dcl2*∆*dcl1*) showed no significant preference for any nucleotide (S2 Fig). Mapping results of the 24,519 reads that were underrepresented by at least log_2_≤-1 in ∆*dcl2*∆*dcl1* compared to ∆*ku70*FRT2, were regarded as Dicer-dependent unique reads and revealed a different composition compared to the complete unique read dataset (Fig 1B and Fig 3C). The proportion of reads that mapped to exonic regions had increased (+13.7%), while the amount of rRNAs fragments had strongly decreased (-22.6%). The high percentage of reads originate from intergenic region did not show any significant change (-1.6%). These results led us conclude that the majority of Dicer-dependent sRNAs are processed from RNA molecules that are transcribed from intergenic, and exonic regions, especially from those in antisense orientation. Comparative analyses of the sRNA-producing loci from ∆*ku70*FRT2 and ∆*dcl2*∆*dcl1* showed that the accumulation of sRNAs in 661 sRNA-producing loci were affected by the Dicer-double deletion. In total, 368 loci that produced sRNAs in both orientations and 293 loci that produced sRNA in either sense or antisense orientation were found in ∆*ku70*FRT2, but were no longer present in ∆*dcl2*∆*dcl1*. We further analyzed the distribution of these 661 Dicer-dependent sRNA loci. Within the 368 Dicer-dependent sRNA-producing loci that generated sense and antisense sRNAs, 246 were assigned to coding sequences and 121 to intergenic regions. The origin of the loci that showed a strand-specific accumulation of Dicer-specific sRNAs was determined, too. Of these 293 loci, 123 were assigned to exonic regions in sense and 35 in antisense orientation. Further, 131 were located in intergenic regions and 4 in tRNA genes, respectively (Fig 3D).

**Fig 4:**
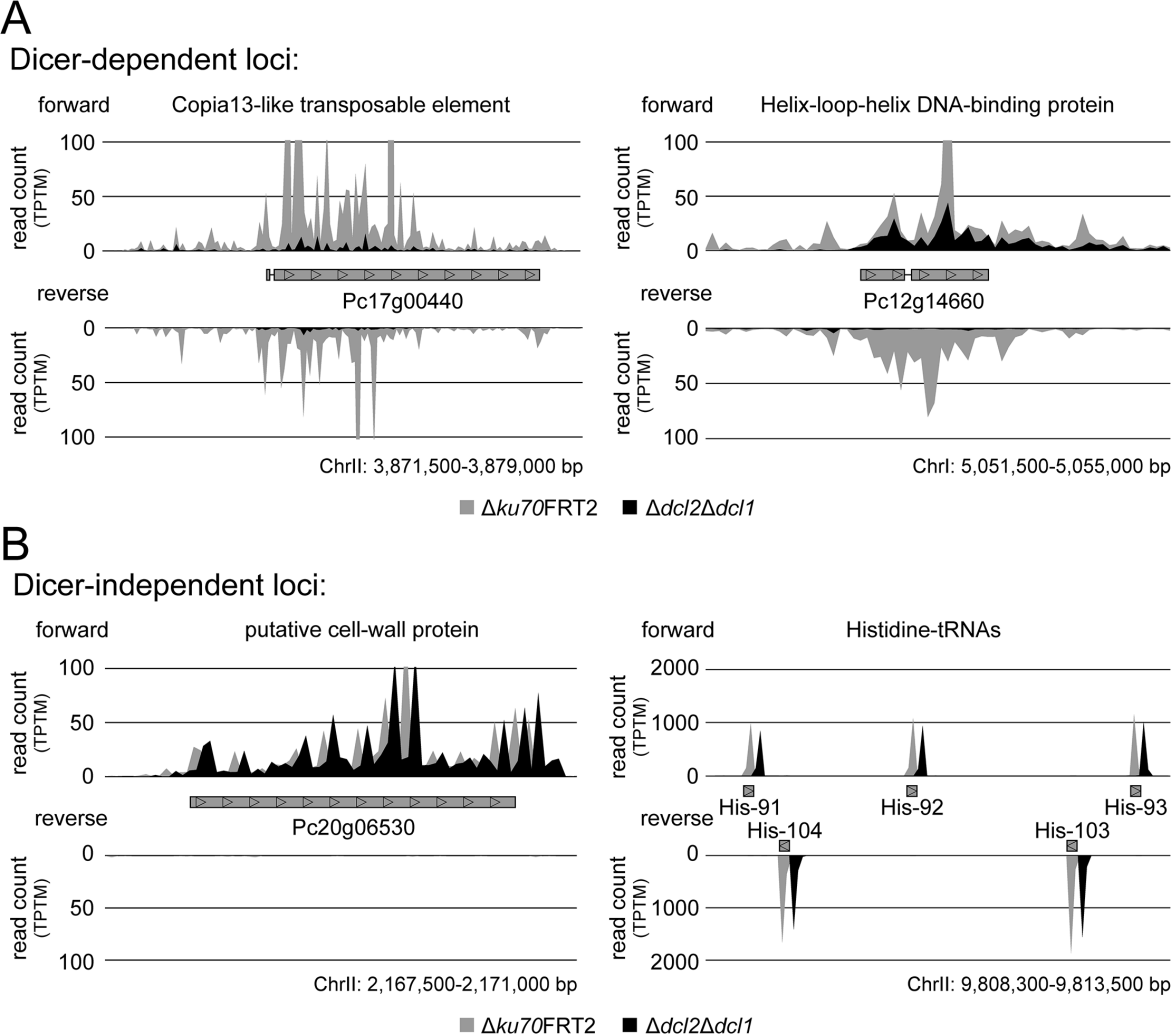
Accumulation of sRNAs along representative coding-sequences. (A) Normalized read count (TPTM: transcripts per ten million) of ∆*ku70*FRT2 (grey graph) and ∆*dcl2*∆*dcl1* (black graph) of two representative Dicer-dependent coding regions, the Copia13-like transposable element Pc17g00440 and the putative DNA-binding protein Pc12g14660. (B) Dicer-independent sRNA accumulation for the coding region of the putative cell-wall protein Pc20g06530 and for a histidine tRNA-gene cluster. To ensure a faultless representation of Dicer-independent reads the graphs for ∆*dcl2*∆*dcl1* were slightly moved to the right.

For representative genes, the distribution of sRNAs from Dicer-dependent and Dicer-independent loci on both strands are presented in Fig 4. Normalized read counts of the Dicer-dependent loci of the Copia13-like transposable element Pc17g00440 and the putative Helix-loop-helix DNA-binding protein Pc12g14660 are shown in Fig 4A. Besides Pc17g00440, multiple copies of the transposable element PCcopia13 (Pc17g00590, Pc21g00460, Pc22g26000, Pc24g01930, and Pc24g02680) and transposable element PCretro14 (Pc24g01940) were identified as Dicer-dependent loci with sRNAs in sense and antisense orientation. In ∆*dcl2*∆*dcl1*, both strands showed a decreased level of sRNAs from transposable elements, while sRNAs from other exonic regions were only significantly decreased on one strand. A representative example for this is the gene Pc12g14660, coding a putative DNA-binding protein, that show a significantly decreased sRNA level only on the antisense strand (Fig 4A). To illustrate sRNA distribution within Dicer-independent sRNA loci, putative cell-wall protein Pc20g06530 and a histidine-tRNA gene cluster are shown in Fig 4B. For these Dicer-independent loci, only sRNAs in sense orientation were detected. For Pc20g06530, sRNAs and mRNAs occur in similar amounts in both the recipient and double deletion strain ∆*dcl2*∆*dcl1*. For all of the above mentioned examples, qRT-PCRs were performed to validate that a depleted amount of sRNA is not dependent from decreased transcription levels of mRNAs.

### Identification of milRNAs in *P*. *chrysogenum*

The processing of microRNAs from an endogenous RNA hairpin molecule by Dicer-like enzymes is a major feature that differentiates milRNAs from other sRNAs. This feature allowed the *in silico* prediction of milRNAs by mapping sRNAs to afore predicted RNA-hairpin precursors. The position and frequency of sRNAs within the set of precursors was used to calculate milRNAs with miRDeep2 [28,29]. The RNA samples obtained from RNA-Mix and ∆*ku70*FRT2 contain Dicer-dependent sRNAs and were used for milRNA prediction, while sRNAs from ∆*dcl2*∆*dcl1* were used as a negative control. For each dataset, milRNAs were predicted independently.

20 putative milRNAs were found for the RNA-Mix and 22 in ∆*ku70*FRT2. Eight milRNAs were found in both datasets, providing a total of 34 different milRNAs, designated milR-1 to -34. As expected for the Dicer-dependent formation of milRNAs, the number of corresponding reads were significantly reduced in ∆*dcl2*∆*dcl1*. On the contrary, all predicted milRNAs found in ∆*ku70*FRT2 were also present in the RNA-Mix sample, and *vice versa* (Table 3). 31 of the 34 predicted milRNAs had a 5' uracil, which is another indication for Dicer-processing of the predicted milRNAs, and the milRNA precursor sequences differed in length from 43 to 89 nt. The ∆*dcl2*∆*dcl1* dataset helped to exclude predictions of milRNAs that were based on random patterns. Here, only two false-positives were found that did not show the typical features of predicted milRNAs, such as a 5'- uracil or the presence of a milRNA-star sequence.

**Table 3:** Predicted milRNAs and the appearance of their reads within the three datasets.

milRNA	Sequence (5'-3')	Normalized Expression Level (TPTM^[^ ^1^ ^]^)			Score^[^ ^2^ ^]^	MFE^[^ ^3^ ^]^	Precursor location
		RNA-Mix	∆*ku70*FRT2	∆*dcl2*∆*dcl1*			
**milR-1**	uugguacgaucgauuggaga	122	305	1	882.2	-41.0	ChrI: 11548223–11548283, exon sense (Pc16g07970)
**milR-2**	ugagaacgcugauauauauau	152	3	0	373.4	-17.1	ChrIII: 2410668–2410742, intergenic
**milR-3**	uagaagaucaugcagcaugua	144	148	0	356.5	-29.2	ChrI: 13214806–13214889, exon antisene (Pc15g01560)
**milR-4**	ugcgacacaagaacaaucggacgau	6	40	0	208.3	-22.4	ChrIII: 3988218–3988278, exon sense (Pc22g06050)
**milR-5**	ugacauaggaacgacgaga	17	27	0	231.2	-24.2	ChrIV: rev(37091–37159), exon antisense (Pc16g00090)
**milR-6**	ucgggcccguagcugugaugc	69	123	0	167.2	-32.7	ChrI: 11697045–11697111; intergenic
**milR-7**	uagguucaggaaguucucucc	40	12	0	166.2	-25.2	ChrI: rev(11697681–11697758), intergenic; ChrI: rev(12068486–12068563), intergenic; ChrII: rev(4407094–4407171). intergeneic
**milR-8**	uggcuacggauaugacucuc	114	35	0	161.5	-42.6	ChrII: 4498587–4498671, intergenic
**milR-9**	ggccccagagaucguuggucuaa	43	31	0	154.3	-25.9	ChrII: rev(6202677–6202728), intergenic
**milR-10**	uagccaucucgucgagguaga	123	46	4	143.4	-29.9	ChrIII: rev(877512–877577), intergenic
**milR-11**	uaugucugcucugguccaugac	4	12	0	136.9	-49.6	ChrI: 12126475–12126539, exon sense (Pc16g15350)
**milR-12**	uccaggcugacgguggcgca	23	2	0	135.4	-35.1	ChrIV: 2408756–2408823, exon antisense (Pc13g13430)
**milR-13**	uaggacuugaucgcgugagac	12	5	0	128.5	-28.7	ChrI: 8239273–8239341, exon antisense (Pc13g04730)
**milR-14**	uagaaugcaagcgcguugagc	52	27	1	104.6	-24.4	ChrII: rev(3729478–3729558), intergenic
**milR-15**	uucgauugggaucugggccu	28	19	0	91.4	-33.6	ChrI: rev(7068824–7068895), intergenic
**milR-16**	ugggcgggcgagauugaac	66	14	0	90.4	-30.0	ChrI: rev(9237687–9237761), exon sense (Pc13g08790)
**milR-17**	uccaucguggcugugcacuu	19	20	1	75.6	-26.9	ChrII: rev(5605185–5605257), exon antisense (Pc21g03660)
**milR-18**	uggacgaggaacaucugcac	12	1	0	73.0	-25.7	ChrI: rev(8625875–8625949), exon antisense (Pc13g06180)
**milR-19**	cggacgagaaugccgaggcucuguu	20	5	0	67.3	-52.8	ChrIV: 979955–980021, intergenic
**milR-20**	uccuacguuacuccaaaggau	10	1	0	61.9	-29.1	ChrI: 8009736–8009780, intergenic
**milR-21**	ucccccucgagaagaucuagucugccucu	8	1	0	59.2	-34.0	ChrI: 7174825–7174898, intergenic
**milR-22**	uuuggaugaaauucgaaugaa	15	2	0	37.9	-32.8	ChrIII: 1228283–1228364, intergenic
**milR-23**	uagacuguuccaaggaugcu	11	14	1	36.6	-25.0	ChrII: 8010371–8010446, exon antisense (Pc21g13900)
**milR-24**	ugcacaaguggacucucccu	10	4	0	36.6	-21.7	ChrI: 12845073–12845153, exon sense (Pc06g00870)
**milR-25**	ucaucggcaaaacuuggagaa	5	8	0	32.2	-28.5	ChrII: rev(3651114–3651202), intergenic
**milR-26**	ucacacguagaaauccagau	8	6	0	31.6	-19.8	ChrI: 10506362–10506430, intergenic
**milR-27**	ucuaccgagacugucuuuga	13	6	0	31,2	-31.3	ChrI: 4059417–4059459, intergenic
**milR-28**	uuagcaugcaugguauugua	2	13	0	30.8	-34.5	ChrII: 8593139–8593200, intergenic
**milR-29**	ugcuuggucgucacucggga	11	6	0	28.2	-28.4	ChrII: 2351660–2351714, intergenic
**milR-30**	uaccaagucgucgaaaugcu	13	3	0	25.1	-27.1	ChrIV: 190879–190926, exon antisense (Pc16g00780)
**milR-31**	ugagaccgcggagcaaacg	29	12	0	24.0	-24.5	ChrI: 3765153–3765197, intergenic
**milR-32**	uggagaaugucacuuguggaa	2	4	0	22.6	-36.1	ChrIII: rev(719002–719055), exon antisense (Pc12g03150)
**milR-33**	augcccuucggcguuagucuacc	5	1	1	22.4	-31.6	ChrIV: rev(1820276–1820326), intergenic
**milR-34**	uuucccaucucgaucaccgga	15	1	0	20.8	-19.0	ChrIV: rev(1325461–1325507), intergenic

^[1]^ TPTM: transcripts per ten million

^[2]^ Score: miRDeep2 prediction score

^[3]^ MFE: minimal free energy

All 34 milRNA precursors are located outside of tRNA and rRNA genes. The precursor sequence of most milRNAs occurred only once with a perfect match within the genome. Only the precursor sequence of milR-7 was found at three different loci (Table 3). To explore the location of milRNAs within the nuclear genome further, the mature sequence of milRNAs was also mapped to the genome. Twenty-four mature milRNAs mapped only once to the reference genome, and 10 (milR-6, milR-7, milR-8, milR-11, milR-12, milR-13, milR-14, milR-26, milR-27, milR-30) mapped at least twice. The most frequent milRNA sequences are milR-13, and milR-11, with 13 and 8 loci. While milR-13 was found in exons, introns, 5’-UTRs, and 3’-UTRs, all loci of milR-11 were located within the 5' end of the coding sequence of multiple copies of a putative RNA-dependent DNA-polymerase (RdDP). This RdDP was previously identified as the Copia13-like transposable element PCcopia13 [30]. All copies of PCcopia13 have highly complementary loci, including the mature milR-11 and milR-11-star sequence.

### MilRNA target gene prediction

So far, no algorithms are available that have been adapted to fungal milRNAs. Therefore, target predictions were performed using three different programs, written for either animals or plants. The program miRanda was written for accurately detecting microRNA binding sites in animals, and the programs TAPIR and psRNATarget were originally written for predictions in plants [31–33]. Since no 3'-UTR database is available for *P*. *chrysogenum*, we extracted the 1 kb downstream sequences of all protein coding genes in the P2niaD18 genome. These 1 kb sequences were used as templates for target prediction. We succeeded in identifying mRNA targets for the predicted milRNAs described in this report. For 30 milRNAs, at least one target binding site was predicted with at least one program (S1 Table), and for 9 milRNAs (milR-6, milR-7, milR-8, milR-11, milR-14, milR-29, milR-30, milR31, milR-34) the same target sites were confirmed with programs written for the prediction of microRNAs in animals (miRanda) and plants (TAPIR and/or psRNATarget). Because some milRNAs are derived from 3'-UTRs, we compared the target sequence with the corresponding milRNA-star sequence, and only the predicted target sites of milR-8 and milR-14 are limited to their own milRNA-star sequences (Table 4). The largest number of putative target sites was predicted for milR-21; 118 binding sites of milR-21 were found within the 3'-UTRs of *P*. *chrysogenum*. The most interesting mRNA targets were predicted for milR-1 and milR-21. For the most common milRNA, milR-1, the *dcl2* (Pc12g13700) gene was predicted as target. For milR-21 at least three genes that might be involved in the DNA-repair mechanisms were predicted. These interesting findings led us to select milR-1 and milR-21 for further analyses.

**Table 4:** milRNA target gene prediction.

milR ID	target ID	function	miRanda score	TAPIR score	psRNATarget UPE	Comment^[^ ^1^ ^]^
**milR-1**	Pc22g17450	hypothetical protein	164	-	-	-
	Pc12g13700	Dicer-like protein (*dcl2*)	161	-	-	-
	Pc12g02820	hypothetical protein	-	4	-	-
**milR-6**	Pc12g15260	hypothetical protein	174	-	17.2	-
**milR-7**	Pc22g02960	SNARE associated Golgi protein	179	-	17.0	-
**milR-8**	Pc04g00060	hypothetical protein	190	0	21.7	star
	Pc19g00700	hypothetical protein	190	0	23.6	star
	Pc24g01200	hypothetical protein	190	0	23.8	star
	Pc24g02780	hypothetical protein	190	0	24.5	star
**milR-11**	Pc16g15330	hypothetical protein	200	0	20.1	star
	Pc12g06730	hypothetical protein	179	2.5	-	-
**milR-14**	Pc24g01250	hypothetical protein	195	0	13.3	star
	Pc17g00230	hypothetical protein	-	1	14.4	-
	Pc21g00150	putative proline-rich call wall protein	-	2	15.2	-
	Pc24g02850	hypothetical protein	-	2	17.1	-
**milR-21**	Pc21g17740	SNF2 family helicase	290	-	-	-
	Pc12g12880	ubiquitin carboxyl-terminal hydrolase	286	-	-	-
	Pc13g12300	BRCA1-like DNA repair protein	283	-	-	-
	Pc13g05360	putative Ku70-binding protein	-	-	20.8	-
**milR-29**	Pc20g05760	putative cytochrome P450 protein	190	0	18.5	star
	Pc18g04580	hypothetical protein	170	3	19.9	-
**milR-30**	Pc22g16980	hypothetical protein	169	3.5	21.2	-
**milR-31**	Pc22g13440	hypothetical protein	185	0	-	star
	Pc21g05840	putative Zn(2)-C6 DNA-binding protein	176	2.5	-	-
	Pc21g02000	PLC-like phosphodiesterase	171	4	-	-
	Pc12g10520	hypothetical protein	172	3.5	-	-
**milR-34**	Pc16g04680	hypothetical protein	195	0	19.9	star
	Pc18g05650	hypothetical protein	171	-	21.7	-

^[1]^ Predicted target sites were compared to the milRNA-star sequence.

### Expression analysis and experimental validation of milRNAs

**Fig 5:**
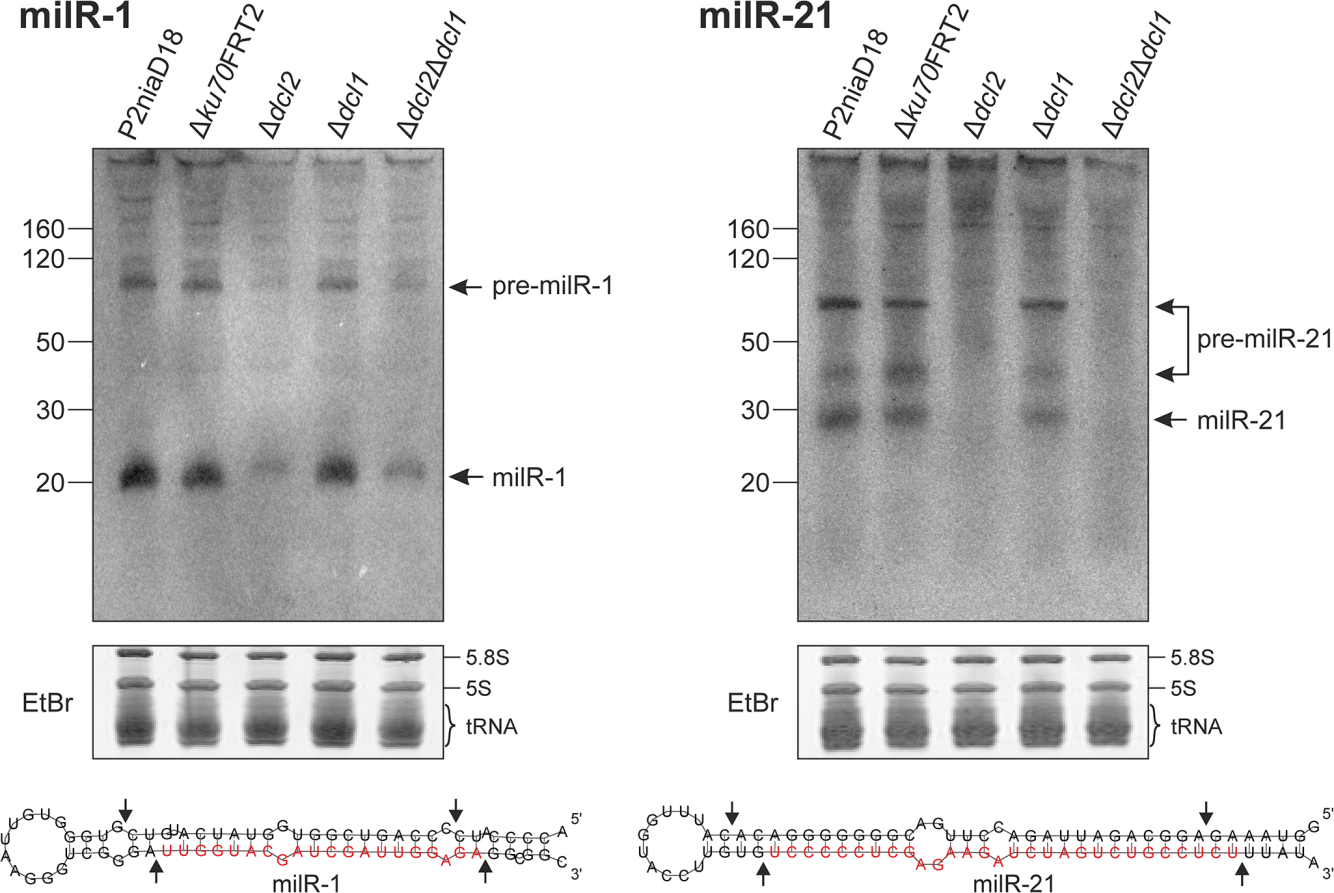
Validation and expression analysis of (A) milR-1 and (B) milR-21. Total RNA from strain P2niaD18, the recipient ∆*ku70*FRT2 as well as Dicer single and double mutant strains were used for polyacrylamide gel electrophoresis and northern blot analysis. Mature milRNAs (milRs) and milRNA precursors (pre-milRs) were detected with revers complement ^32^P-labled DNA probes. Below, loading controls of the total RNA, stained with ethidium bromide (EtBr), and the predicted secondary structures of milRNA precursors are given. On the secondary structures, milRNA sequences are highlighted in red and arrows indicate the expected Dicer cleavage sites.

To confirm the presence of milRNAs in *P*. *chrysogenum*, we performed northern blot analysis to detect milR-1 and milR-21. For northern blotting, total RNA from mycelia grown under the same conditions as for the library construction of ∆*ku70*FRT2 and ∆*dcl2*∆*dcl1* was used to detect predicted milRNAs. Both processed milRNAs as well as their precursor RNAs were detected by radioactive labeled probes (Fig 5). Interestingly, additional signals detected between the precursor and mature milR-21 may represent intermediates of the processed precursors.

To test Dicer-dependent processing of milRNAs, further expression analyses were performed with total RNA from strains lacking one or both Dicer-like proteins. As expected for the *dicer*-double deletion strain, milR-21 and its precursors were not detected. Furthermore, milR-21 and its precursors were also absent in the *dcl2*-deletion, but not in the *dcl1*-deletion strain, indicating that processing of milR-21 is Dcl2-dependent. Interestingly, weak signals for milR-1 and its precursors were detected in the Dicer-deficient strain ∆*dcl2*∆*dcl1* and the ∆*dcl2* single deletion strain. These signals were significantly weaker than the signals observed from strains with an intact *dcl2* gene. This result is consistent with the sRNA sequencing data presented in Table 3.

## Discussion

### Evidence for small RNAs in *P*. *chrysogenum*

We previously reported that in *P*. *chrysogenum* silencing of endogenous genes by artificial siRNAs is Dcl2 dependent [24]. Here we extend our analysis using small RNA deep sequencing of three different samples to increase our knowledge about endogenous fungal sRNAs, and specifically to identify microRNA-like RNAs. Indeed, we identified 34 milRNAs in *P*. *chrysogenum* and demonstrate that the majority of sRNAs, including the novel milRNAs are processed from RNA molecules transcribed from intergenic and exonic regions, especially those in antisense orientation. This agrees with a previously study in the ascomycete *Fusarium oxysporum*, where intergenic regions provided a high proportion of sRNAs [16]. In *Magnaporthe oryzae* it was further demonstrated that antisense sequences of coding regions can provide numerous sRNAs [34]. Further, a class of endogenous sRNAs that map to exons, called exonic-siRNAs, regulate the expression of protein coding genes in the basal fungus *Mucor circinelloides* [35]. To avoid analysis of mRNA degradation products, we used a strict cut-off for unique reads and identified sRNA-producing loci. We identified 661 Dicer-dependent sRNA-producing loci and demonstrate that 368 of these loci generate sRNAs in sense and antisense orientation from the same genomic source. The number of loci however that generate sRNA in both orientations is significantly decreased in the Dicer-deficient double-mutant. Further Dicer-dependent sRNAs are obtained mostly originating from intergenic and exonic regions in sense and antisense orientation. This observation is in accordance with the Dicer-dependent cleavage of dsRNAs and studies of exonic-sRNAs in *M*. *circinelloides* [35].

Although no obvious phenotype has so far been detected in ∆*dcl2*∆*dcl1*, we clearly demonstrated a lack of Dicer-dependent sRNAs in the Dicer-double mutant. Further, we showed that the amount of sRNAs on both strands of the selfish genetic element PCcopia13 (Pc17g04400) was significantly reduced in the Dicer-deficient double-mutant. This observation is consistent to results obtained for transposons from *Neurospora crassa* and *Magnaporthe oryzae* and for the antiviral defense mechanism in the chestnut blight fungus *Cryphonectria parasitica* [36–38]. Genetic stability is an essential feature for industrial applications using *P*. *chrysogenum* [39]. Therefore, a well-functioning defense mechanism against selfish transposable elements is an important requirement of industrial strains to guarantee stable production yields.

Further, we observed typically characteristics for sRNAs known from other filamentous fungi, like significant preference for a 5'-uracil, their length distribution, and their origin from intergenic and exonic regions [40]. However, the percentage of uracil-starting Dicer-dependent sRNAs was only drastically higher for sRNAs ranging from 17 to 23 nt. Nevertheless, Dicer-dependent sRNAs were the predominant class of sRNAs in the overall sRNA population. We have demonstrated that formation of milR-1 is only partially Dicer-dependent. This result infers that at least one Dicer-independent pathway may be present to generate milR-1 in the Dicer-deficient mutant. This idea is strengthened by the presence of various Dicer-independent pathways of interfering sRNA in *N*. *crassa* [12]. Therefore, further analyses are necessary to reveal Dicer-independent sRNA-biogenesis pathways in *P*. *chrysogenum*.

### Evidence for milRNAs

Our bioinformatics analysis identified 34 Dicer-dependent sRNAs that carry the typical features of conventional miRNAs as similarly characterized in other eukaryotes. Typical features are RNA stem-loop formation, a strong preference for uracil at the 5' end, and an average size of about 21 nt [5,41]. We predicted 8 milRNAs in the small RNA sequencing datasets of both RNA-Mix and ∆*ku70*FRT2, and repeatedly deriving these 8 milRNAs demonstrates the reliability of our analysis. Due to different growing conditions, some milRNAs were only predicted in the RNA-Mix or ∆*ku70*FRT2 dataset. Nevertheless, all mature milRNAs were represented by reads within both datasets. This indicates different expression rates of milRNA under different growth conditions. Further, these reads were either totally absent or reduced to single reads in the ∆*dcl2*∆*dcl1* double mutant.

Since knowledge about fungal milRNAs is still limited, improving parameters to predict fungal milRNAs is a challenging task for the future. However, we were able to identify Dcl2-dependent milRNAs ranging in size between 19 and 29 nt, which is consistent with previous findings that some mature fungal milRNAs are longer than regular animal and plant miRNAs [12,16]. Nevertheless, the majority of fungal Dicer-dependent sRNAs and milRNAs range in size between 19 and 22 nt. All predicted mature milRNA sequences of *P*. *chrysogenum* were compared to those recently found in other fungi, but no conserved milRNAs were found.

The predicted precursor sequences range in size between 43 to 89 nt. These results are consistent with milRNAs and milRNA-precursors found for other ascomycetes [12,14–16]. Northern blot analyses confirmed the existence of precursor and mature milRNAs milR-1 and milR-21 in *P*. *chrysogenum*. So far, we were unable to detect pri-milRNAs precursors, most probably due to their low abundance. When analyzed in detail, both milRNAs proved to be complete or partial Dcl2 dependent; moreover Dcl1 and Dcl2 did not show functional redundancy. These observations are also supported by our previous analyses demonstrating that only Dcl2 is essential for processing artificial dsRNAs to generate functional siRNAs that silence genes containing a perfect complementary target site [24]. These results are consistent with findings of milRNA biogenesis in *P*. *marneffei* but in contrast to findings in *N*. *crassa*, where both Dicer-like proteins are functionally redundant [12,17,42].

Remarkably, milR-1 was detected in the Dicer double-deletion strain. This demonstrates that milR-1 can form Dicer-independently, and that an alternative Dicer-independent pathway may exist to generate milRNAs. In *N*. *crassa* at least four different milRNA-generating pathways have been discovered involving components of Dicer-like proteins, Argonaute proteins, exonucleases (QIP) and an RNase III domain-containing protein (MRPL3). It was also demonstrated that single milRNAs can be processed independently by two different pathways [12].

Here, we clearly demonstrate that in *P*. *chrysogenum* the majority of sRNAs are Dicer-dependent and that Dcl2 is essential for the maturation of milR-21 and necessary for the accumulation of milR-1. Since the majority of predicted milRNAs are not present in Dicer double-deletion strain ∆*dcl2*∆*dcl1*, their generation is Dicer dependent.

Among the predicted 34 milRNAs, 24 map only once to the reference genome, and 10 at least twice. Surprisingly, one of the most abundant milRNA sequences was milR-11, found 8 times in the 5' end of the coding sequence for the Copia13-like transposable element PCcopia13. We demonstrated that the level of sRNAs from this transposable element was reduced on both strands in ∆*dcl2*∆*dcl1*. This indicates strongly to an sRNA-mediated RNAi pathway of selfish genetic elements in *P*. *chrysogenum*. The location of milRNA genes inside transposable elements is consistent with recent studies in *Cryptococcus neoformans*, where multiple loci of two milRNAs were discovered in the sequences of transposable elements and pseudogenes. It was demonstrated that milRNAs in *C*. *neoformans* induce transgene silencing via the canonical RNAi pathway [13]. Our results prompt us to believe that milRNAs in *P*. *chrysogenum* are likewise involved in the negative regulation of transposon activity.

### Various mRNA targeting by milRNA?

Using diverse programs, we were able to predicted potential milRNA target sites for all 34 milRNAs on putative 3'-UTRs of *P*. *chrysogenum*. Eight milRNA binding sites, calculated by miRanda, were validated by the plant miRNA-prediction programs TAPIR and/or psRNATarget. While some milRNAs had only a single predicted target, others, like milR-21, have the option of interacting with multiple mRNA targets. Interestingly the second best predicted target for milR-1 is the *dcl2* gene (Pc12g13700) und milR-21 was predicted to bind to at least three mRNA targets that are involved in DNA-repair mechanisms. As yet, we were not able to detect milRNA-mediated mRNA-degradation in *P*. *chrysogenum*. Previously, reporter gene constructs with artificial target sites were used to demonstrate milRNA-mediated silencing in fungi [12,13]. In these studies, target-mRNA degradation has only been demonstrated with reporter genes that carried a fully complementary target site. In contrast to siRNAs, miRNAs do not require full complementarity to bind and mediate silencing of target mRNAs. Thus, it is suggested that milRNA-mediated gene silencing in fungi is mostly caused by translational repression like it is known for animals and plants [1,12,43]. Therefore, further analyses of target-mRNA protein expression are necessary provide evidence for milRNA-target interactions and thus, milRNA mediated gene silencing in *P*. *chrysogenum*. Nevertheless, our small RNA sequencing approach identified novel milRNAs that are predicted to bind multiple target mRNAs, possibly as factors involved in a variety of different biological processes, like DNA-repair. Finally, results from sRNA identification and milRNA target predictions will serve as valuable clues for understanding sRNAs as regulators of gene expression in *P*. *chrysogenum*. A deeper understanding of sRNA-mediated gene regulation is an important prerequisite for developing more effective strategies to genetically manipulate this industrially important fungus to further improve production processes.

## Materials and Methods

### Strains and growth conditions

For the RNA-Mix, *P*. *chrysogenum* strain P2niaD18 was grown as submerged cultures in 500 ml shaking flasks in 100 ml CCM and MM at 27°C and 120 r.p.m., and as surface cultures on PAL Science Supor 200 Disc Filters on solid Oatmeal and M322 media. All cultures were inoculated with 1.0 x 10^7^ conidiospores as previously described [44,45]. Liquid cultures were grown for 72, 96, and 120 hours, and surface cultures for 48, 72, and 96 hours. The mycelia of these 12 different cultures were frozen in liquid nitrogen and total RNA was isolated separately as described in the "Nucleic acid isolation" section. After purification, total RNA of the 12 samples were pooled in equal amounts to the so-called RNA-Mix sample. For the samples ∆*ku70*FRT2 and ∆*dcl2*∆*dcl1* the recipient strain ∆*ku70*FRT2 and the Dicer-deficient double-mutant ∆*dcl2*∆*dcl1* were grown for three days in shaking flasks containing CCM under the same conditions as mentioned above (S2 Table and S3 Table).

### Identification of RISC core components

The recently published genome of *P*. *chrysogenum* strain P2niaD18 [25] and the previously published annotation of strain Wisconsin 54–1255 [46] served as source for identifying genes involved in RNAi. To identify core components, BLAST analyses of RISC proteins from *N*. *crassa* were performed (S3 Fig and S4 Table) [47]. Protein sequence data from *N*. *crassa* were obtained from the National Center for Biotechnology Information (NCBI) (http://www.ncbi.nlm.nih.gov/), and sequences were aligned using MAFFT [48]. For domain identification, the predicted *P*. *chrysogenum* protein sequences were used to perform an NCBI conserved domain search (http://www.ncbi.nlm.nih.gov/Structure/) [49].

### Generation of Dicer single- and double-knockout strains

Plasmids were constructed to substitute genes encoding Dicer-like proteins in *P*. *chrysogenum* recipient strain ∆*ku70*FRT2 (S5 Table). For homologous recombination, fragments of about 1 kb flanking the 5' and 3' ends of the coding sequence were amplified by PCR and ligated to corresponding 5' and 3' flanks of nouseothricin or phleomycin knockout cassettes, expressing the *nat* or *ble* gene, under control of the *trpC* promoter. For deletion of *dcl2* (Pc12g13700), the gene was replaced with a phleomycin resistance cassette. The *nat*-knockout cassette, used for deletion of *dcl1* (Pc21g06890), carries additional components of the *FLP*/FRT recycling system that are under the control of the inducible promoter *xylP* [50]. Transformation of *P*. *chrysogenum* strains with foreign DNA was performed using conventional transformation procedures with modifications as previously described [45,51]. Homologous integration of the knockout construct and substitution of the target genes were confirmed by Southern blot and PCR analysis covering regions within and flanking the knockout cassette (S4 Fig, S5 Fig and S6 Fig). With this procedure, we generated *dcl1-* and *dcl2*-deletion strains. Transformation of plasmid pKOdcl1 in *dcl2*-deletion strain resulted finally in the construction of the ∆*dcl2*∆*dcl1*double-deletion mutant.

### Nucleic acid isolation

As described in the "Strains and growth conditions" sections, total RNA used for small RNA sequencing was obtained from different cultivations. For the "RNA-Mix" sample, total RNA was isolated independently from twelve different cultures of strain P2niaD18. For samples ∆*ku70*FRT2 and ∆*dcl2*∆*dcl1* a single culture of the corresponding strains was grown in liquid CCM for 72 h. For all samples, mycelia were collected for guanidinium-thiocyanate-phenol-chloroform extraction of total RNA [52]. RNA was measured with NanoDrop 1000 Spectrophotometer and Bioanalyzer 2100, using the Agilent RNA 6000 Nano Kit. All total RNAs that were pooled to equal amounts for the RNA-Mix show RNA integrity numbers (RIN) ≥ 9.0. For the analysis of ∆*ku70*FRT2 and ∆*dcl2*∆*dcl1* only total RNA with RIN values ≥ 9.8 were used. The 28S:18S ratio was calculated with 1.7 for all samples. DNA preparation was carried out as described recently [53].

### Small RNA deep sequencing

Total RNAs from the three different samples described above in the “Strains and growth conditions” section were used for small RNA library preparation and small RNA sequencing. Library preparation and sequencing was performed at GATC Biotech, Konstanz, Germany. Total RNA samples were separated by a 10% TBE-urea denaturing polyacrylamide gel electrophoresis (PAGE) and sRNAs ranging between 18 and 50 nt were used for library preparation using the Illumina TruSeq Small RNA Sample Preparation Kit. After reverse transcription and amplification, cDNA products were checked and measured with Bioanalyzer 2100. Sequencing was performed on an Illumina HiSeq 2000 platform. Trimming of TruSeq adapter sequences was performed with the program Cutadapt and only reads trimmed at their 3’ end were used for further studies [54]. Raw sequencing data from sRNA sequencing have been deposited in the NCBI sequence read archive (SRA) (http://www.ncbi.nlm.nih.gov/sra) under the accession numbers SRR1705825 (RNA-Mix), SRR1706009 (∆*ku70*FRT2), and SRR1706010 (∆*dcl2*∆*dcl1*).

### Small RNA data analysis and milRNA prediction

To examine the origin of the cleaned RNA reads, alignments against the reference genome of *P*. *chrysogenum* P2niaD18 and diverse datasets, containing all intronic, exonic, and intergenic regions, as well as tRNA and rRNA sequences were performed with bowtie v1.1.0 and only perfect matches were considered [25,55]. This procedure was performed for total reads and unique reads with a read count of at least 10 that were collapsed using the perl script mappler.pl, a part of miRDeep2 [29]. For the calculation of read length and starting nucleotide distribution custom perl scripts were written. To remove spurious sRNAs, derived by RNA degradation, we adapted a previously published method [56]. All unique overlapping or closely mapped sRNAs were grouped. Only loci with at least four unique reads within a sliding interval of 300 bp were taken into account for further analyses. To identify Dicer-dependent reads and loci, unique reads and loci obtained from ∆*ku70*FRT2 and ∆*dcl2*∆*dcl1* were compared to each other. Read counts were normalized (transcripts per ten million, TPTM) using all reads that map to intragenic and intergenic regions. Reads mapping to structural RNAs, like tRNAs or rRNAs, were not considered for normalization. After normalization, log_2_ ratios were calculated using the count number of all unique reads obtained from the Dicer-deficient mutant and ∆*ku70*FRT2. To distinguish between Dicer-dependent and-independent loci, all loci in ∆*ku70*FRT2 and ∆*dcl2*∆*dcl1* were compared in accordance to their strand bias. A genomic region that showed sRNAs either on one strand or in forward and reverse orientation was rated as a sRNA-producing locus. Loci that appeared in ∆*ku70*FRT2 but not in the Dicer-deficient double-mutant ∆*dcl2*∆*dcl1* were assigned as Dicer-dependent.

MilRNAs were predicted with the program miRDeep2 with a score cutoff of 20 and without using additional miRNA information. For all predicted milRNAs, the read counts inside all three unique datasets were determined. For visualization of predicted milRNA precursors, RNAfold, part of the Vienna RNA Package 2.0, was used to predict secondary structures [57].

### MilRNA target prediction

Little is known about milRNA-target interactions in fungi. To include both animal and plant surveys into our analysis, the program miRanda, written to predict miRNA targets in animals, and the programs TAPIR and psRNATarget, to predict potential plant-like target interactions, were used with default settings [31–33]. Since no 3'-UTR database is available for *P*. *chrysogenum* the 1 kb downstream sequences were extracted from all protein coding genes. These hypothetical 3'-UTRs were used for milRNA target prediction.

### Northern blot analyses

For northern blot analysis, conducted to confirm the existence of predicted milRNAs, 30 μg of total RNAs were separated on a 15% denaturing polyacrylamide gel, containing 7 M urea. Separated RNAs were transferred onto a Hybond-N^+^ hybridization membrane (GE Healthcare Life Sciences) and crosslinked by UV-radiation (Stratalinker). Synthesized DNA StarFire probes (DNA Integrated Technology), which were perfectly complementary to the mature milRNA were labeled with α-[^32^P] dATP, following the manufacturer's instructions. Hybridization in modified Church-Gilbert-hybridization solution (0.25 M KH_2_PO_4_, 0.25 M K_2_HPO_4_, 7% SDS, and 1 mM EDTA) was started at 65°C for 1 h, followed by an overnight stepwise cooling phase ending at 35°C [58,59]. For detection of bound radioisotopes Fujifilm BAS-III PhosphorImager plates and FLA-3000 detection system were used.

### Quantitative real-time PCR

Quantitative real-time PCR (qRT-PCR) was carried out in a StepOnePlus real-time PCR system (Applied Biosystems) with GoTaq qPCR MasterMix for SybrGreen (Promega) as described previously [60,61]. Oligonucleotide primers are given in S6 Table.

## Supporting Information

S1 Fig Annotation of small RNA loci of all three datasets, RNA-Mix, ∆*ku70*FRT2, and ∆*dcl2*∆*dcl1*. Pie graphs show the proportion of small RNAs mapped to intergenic, exonic and intronic regions or rRNA and tRNA genes, for total reads and unique reads.
(TIF)

S2 Fig Nucleotide preference of Dicer-dependent and-independent small RNAs. Beside the strong preference for uracil at the 5'-end for Dicer-dependent reads, no further nucleotide preference was detected for other positions inside Dicer-dependent and-independent sRNAs.
(TIF)

S3 Fig Comparative analysis of the Dicer-protein domains of DCL-2 and DCL-1 in *N*. *crassa* and Dcl2 (Pc12g13700) and Dcl1 (Pc21g06890) in *P*. *chrysogenum*. Conserved domains within the proteins are indicated and alignments are displayed below.
(TIF)

S4 Fig Construction and validation of ∆*dcl2* mutants. Replacement of the gene coding for Dcl2 (Pc12g013700), with phleomycin resistance cassette containing the *Streptoalloteichus hindustanus* (Sh) *ble* gene. Validation of homologous integration within the recipient strain ∆*ku70*FRT2 was performed by Southern blotting of 20 μg *Eco*RV digested genomic DNA with ^32^P-labled complementary DNA probes of the 5'- (dark bar) and 3'-flank (bright bar) of *dcl2*. GeneRuler DNA Ladder (Thermo Scientific) was used as size standard. Homokaryotic transformants that show the expected fragments according to a correct genomic integration of the resistance cassette are marked with an asterisk.
(TIF)

S5 Fig Construction and validation of ∆*dcl1* mutants. Replacement of the gene coding for Dcl1 (Pc21g06890), with the inducible *FLP*/FRT cassette containing an N-acetyltransferase coding gene (*nat*) that mediates resistance to the antibiotic nourseothricin. Validation of homologous integration within the recipient strain ∆*ku70*FRT2 was performed by Southern blotting of 20 μg *Bgl*I digested genomic DNA with ^32^P-labled complementary DNA probes of the 5'-flank (dark bar) of *dcl1*. Furthermore, PCR analyses, with primers (indicated with grey arrows) surrounding the sequences used for homologous integration, validate the homologous integration of the resistance cassette. In contrast to the tested transformants, the no-template controls (-Control) and PCRs with the recipient DNA (WT) show no PCR product. GeneRuler DNA Ladder (Thermo Scientific) was used as size standard. Homokaryotic transformants that show correct genomic integrations are marked with an asterisk.
(TIF)

S6 Fig Construction of ∆*dcl2*∆*dcl1* double mutants based on the strain ∆*dcl2*. Homologous integration of the *dcl1* knockout construct resulted in the replacement of the *dlc1* coding gene (Pc21g06890) with a nourseothricin resistance cassette. Validation of homologous integration within the recipient strain ∆*dcl2* T1 was performed by Southern blotting and PCR of 20 μg *Bam*HI digested genomic DNA with ^32^P-labled complementary DNA probes of the 5'-flank (dark bar) of *dcl1*. Furthermore, PCR analyses, with a primer pair (indicated with grey arrows) surrounding the 3'-flank, which was used for homologous integration, validate the homologous integration of the resistance cassette. In contrast to the tested genomic DNA of ∆*dcl1* T1 (+Control), the no-template controls (-Control) and PCRs with the recipient DNA (WT) show no PCR product. GeneRuler DNA Ladder (Thermo Scientific) was used as size standard. Homokaryotic transformants that show correct genomic integrations are marked with an asterisk.
(TIF)

S1 Table Detailed list of predicted milRNA target sites. (XLSX)

S2 Table Strains used in this study. (XLSX)

S3 Table Cultivations used for RNA-Seq samples. (XLSX)

S4 Table Components involved in small RNA biogenesis of *Penicillium chrysogenum* compared to five other fungi. (XLSX)

S5 Table List of plasmids used in this study. (XLSX)

S6 Table List of oligonucleotides used in this study. (XLSX)
