# Supplementary figures and images for: Dicer-Dependent Biogenesis of Small RNAs and Evidence for MicroRNA-Like RNAs in the Penicillin Producing Fungus Penicillium chrysogenum
Source: PLoS One. 2015 May 8;10(5):e0125989. doi: 10.1371/journal.pone.0125989 (PMC4425646; doi:10.1371/journal.pone.0125989)

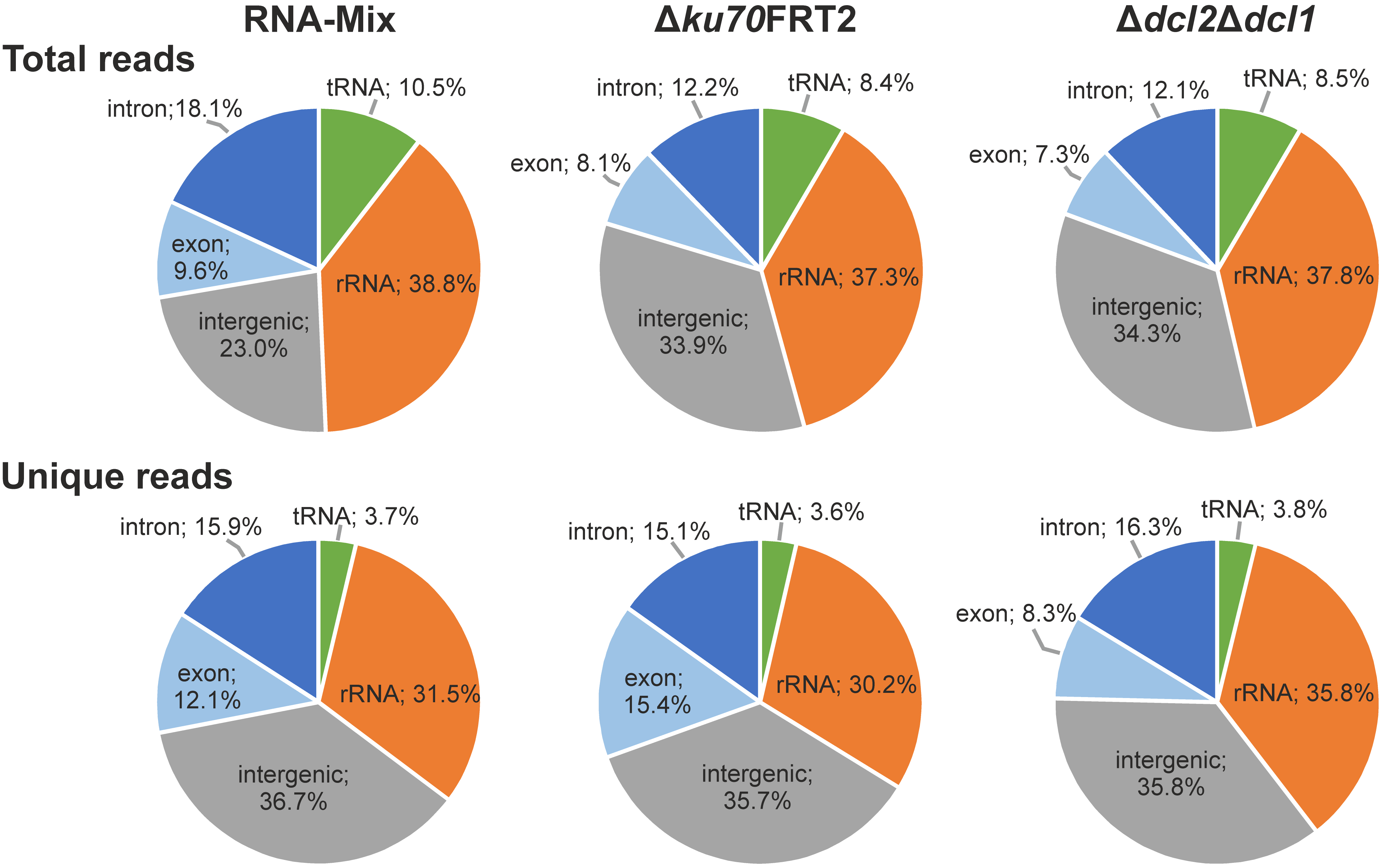

Supplement: S1 Fig — Pie graphs show the proportion of small RNAs mapped to intergenic, exonic and intronic regions or rRNA and tRNA genes, for total reads and unique reads. (TIF) [file pone.0125989.s001.tif]

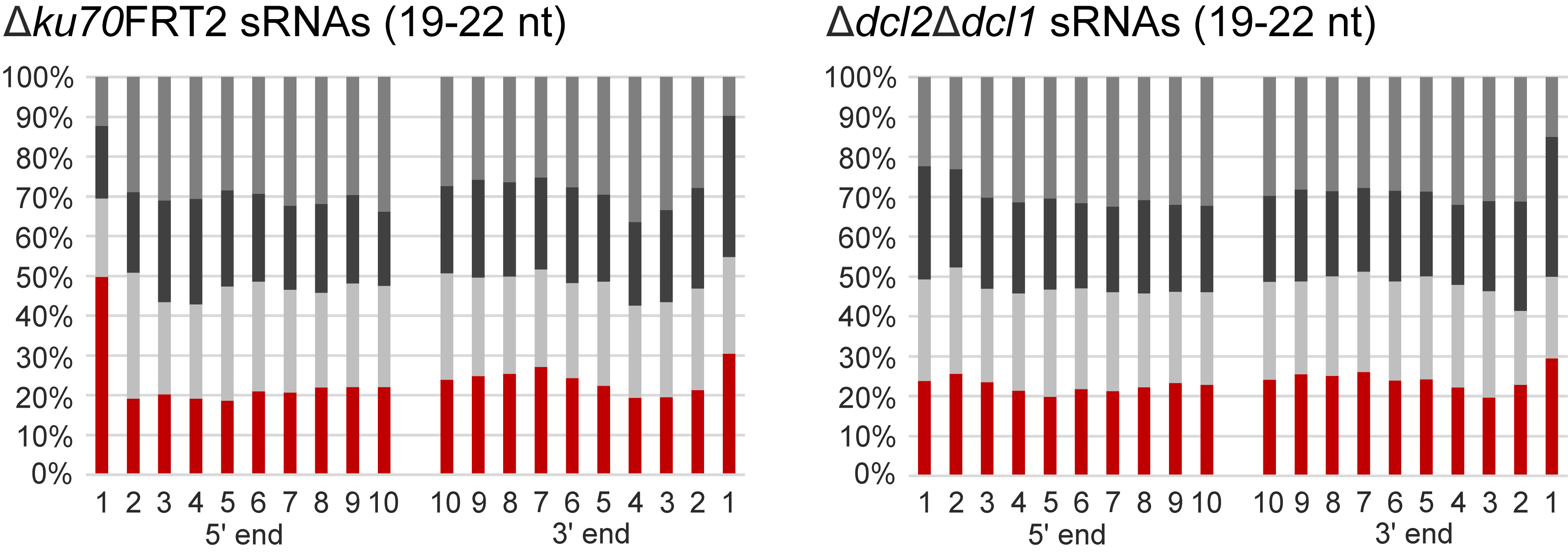

Supplement: S2 Fig — Beside the strong preference for uracil at the 5'-end for Dicer-dependent reads, no further nucleotide preference was detected for other positions inside Dicer-dependent and-independent sRNAs. (TIF) [file pone.0125989.s002.tif]

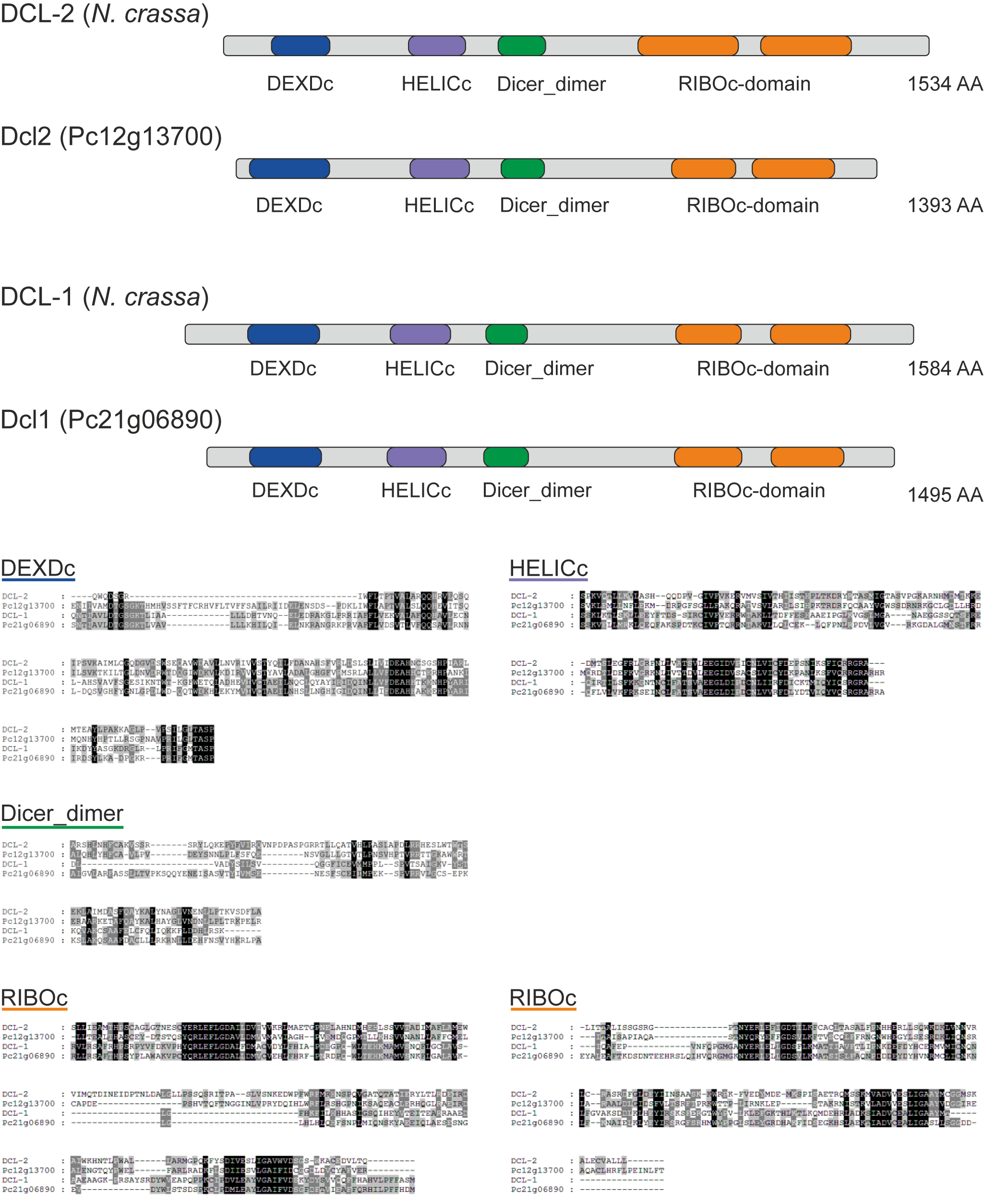

Supplement: S3 Fig — Conserved domains within the proteins are indicated and alignments are displayed below. (TIF) [file pone.0125989.s003.tif]

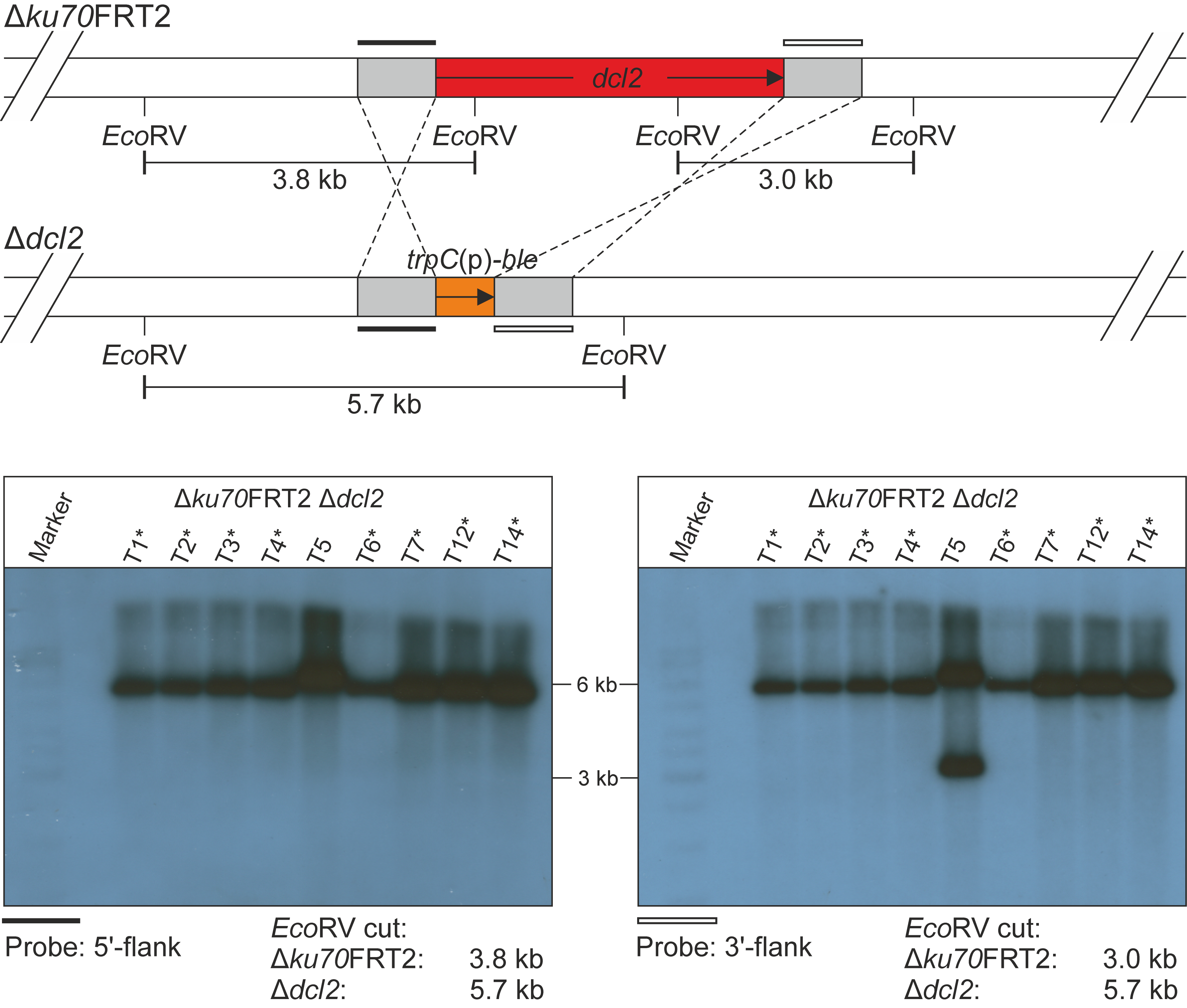

Supplement: S4 Fig — Replacement of the gene coding for Dcl2 (Pc12g013700), with phleomycin resistance cassette containing the Streptoalloteichus hindustanus (Sh) ble gene. Validation of homologous integration within the recipient strain ∆ku70FRT2 was performed by Southern blotting of 20 μg EcoRV digested genomic DNA with 32P-labled complementary DNA probes of the 5'- (dark bar) and 3'-flank (bright bar) of dcl2. GeneRuler DNA Ladder (Thermo Scientific) was used as size standard. Homokaryotic transformants that show the expected fragments according to a correct genomic integration of the resistance cassette are marked with an asterisk. (TIF) [file pone.0125989.s004.tif]

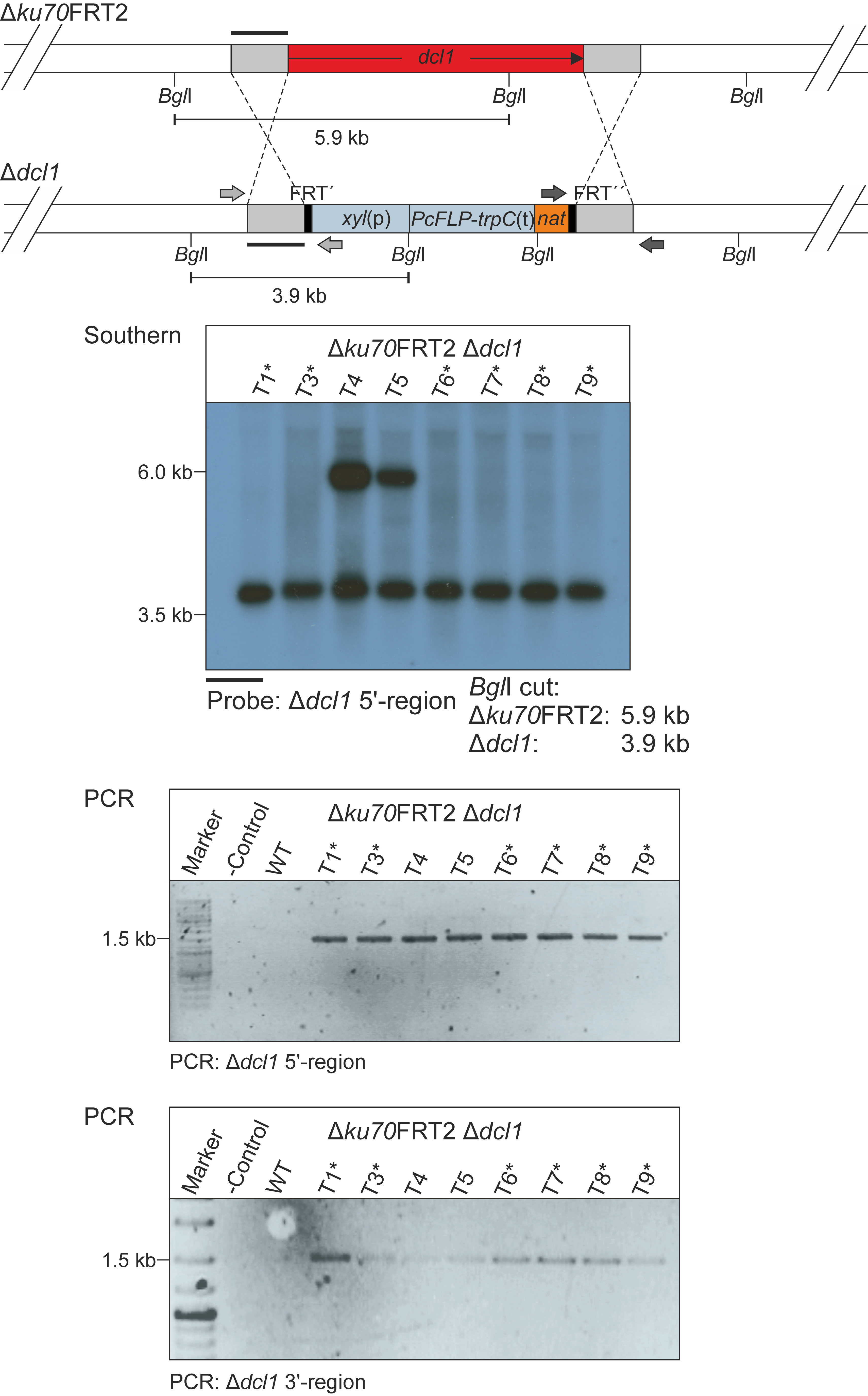

Supplement: S5 Fig — Replacement of the gene coding for Dcl1 (Pc21g06890), with the inducible FLP/FRT cassette containing an N-acetyltransferase coding gene (nat) that mediates resistance to the antibiotic nourseothricin. Validation of homologous integration within the recipient strain ∆ku70FRT2 was performed by Southern blotting of 20 μg BglI digested genomic DNA with 32P-labled complementary DNA probes of the 5'-flank (dark bar) of dcl1. Furthermore, PCR analyses, with primers (indicated with grey arrows) surrounding the sequences used for homologous integration, validate the homologous integration of the resistance cassette. In contrast to the tested transformants, the no-template controls (-Control) and PCRs with the recipient DNA (WT) show no PCR product. GeneRuler DNA Ladder (Thermo Scientific) was used as size standard. Homokaryotic transformants that show correct genomic integrations are marked with an asterisk. (TIF) [file pone.0125989.s005.tif]

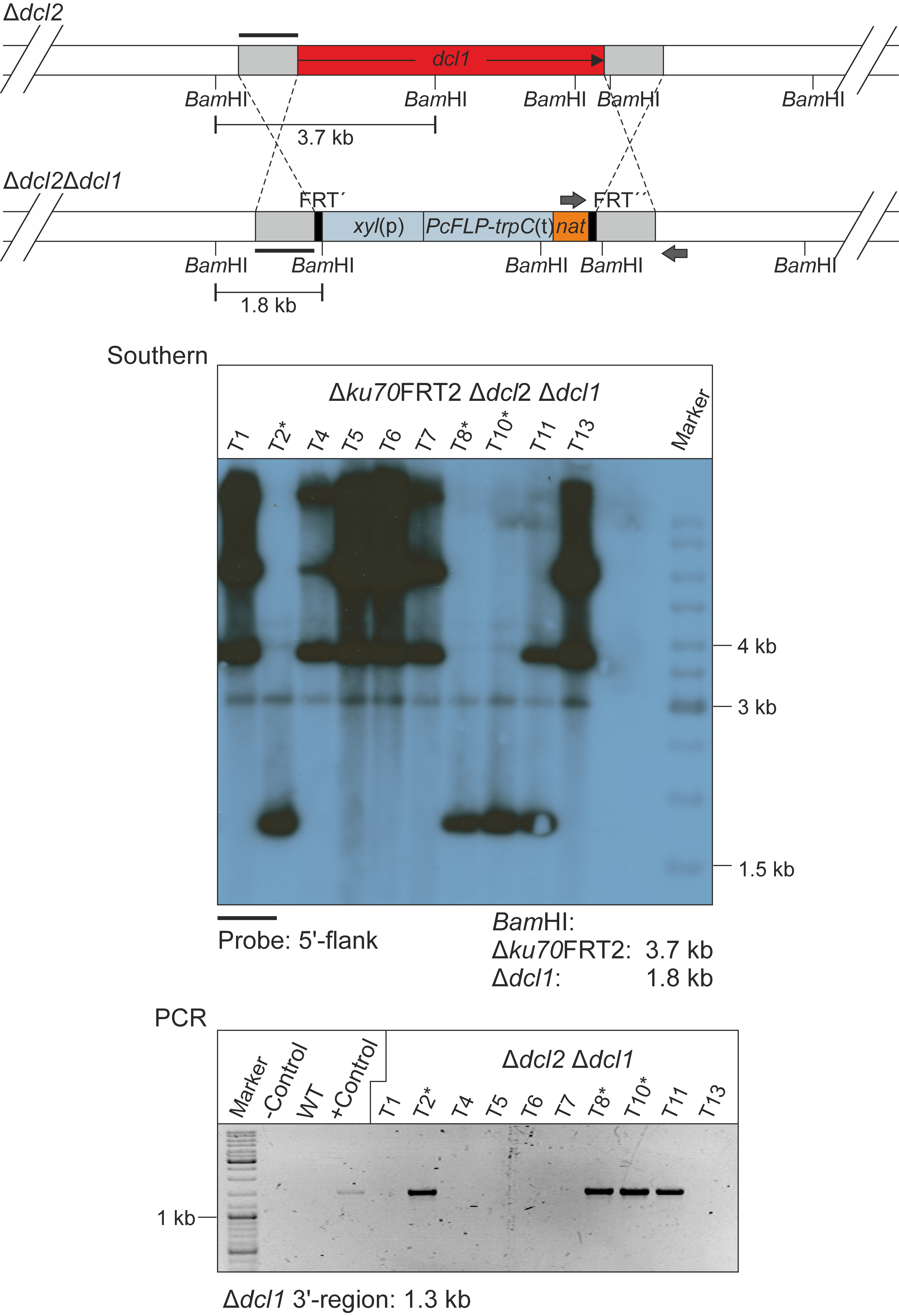

Supplement: S6 Fig — Homologous integration of the dcl1 knockout construct resulted in the replacement of the dlc1 coding gene (Pc21g06890) with a nourseothricin resistance cassette. Validation of homologous integration within the recipient strain ∆dcl2 T1 was performed by Southern blotting and PCR of 20 μg BamHI digested genomic DNA with 32P-labled complementary DNA probes of the 5'-flank (dark bar) of dcl1. Furthermore, PCR analyses, with a primer pair (indicated with grey arrows) surrounding the 3'-flank, which was used for homologous integration, validate the homologous integration of the resistance cassette. In contrast to the tested genomic DNA of ∆dcl1 T1 (+Control), the no-template controls (-Control) and PCRs with the recipient DNA (WT) show no PCR product. GeneRuler DNA Ladder (Thermo Scientific) was used as size standard. Homokaryotic transformants that show correct genomic integrations are marked with an asterisk. (TIF) [file pone.0125989.s006.tif]
